# Advanced fractional soliton solutions of the Joseph–Egri equation via Tanh–Coth and Jacobi function methods

**DOI:** 10.1038/s41598-025-19481-x

**Published:** 2025-10-13

**Authors:** Khadija Shakeel, Dumitru Baleanu, Muhammad Abbas, Majeed Ahmad Yousif, Pshtiwan Othman Mohammed, Farah Aini Abdullah, Thabet Abdeljawad

**Affiliations:** 1 Department of Mathematics, University of Sargodha, Sargodha, 40100 Pakistan; 2 Department of Computer Science and Mathematics, Lebanese American University, Beirut, 11022801 Lebanon; 3 Department of Mathematics, College of Education, University of Zakho, Duhok, 42001 Iraq; 4 Department of Mathematics, College of Education, University of Sulaimani, Sulaymaniyah, 46001 Iraq; 5 Research Center, University of Halabja, Halabja, 46018 Iraq; 6 Research and Development Center, University of Sulaimani, Sulaymaniyah, 46001 Iraq; 7 School of Mathematical Sciences, Universiti Sains Malaysia, 11800 Penang, Malaysia; 8 Department of Fundamental Sciences, Faculty of Engineering and Architecture, Istanbul Gelisim University, Avcılar-Istanbul, 34310 Turkey; 9 Department of Mathematics and Sciences, Prince Sultan University, P.O. Box 66833, 11586 Riyadh, Saudi Arabia; 10 Department of Medical Research, China Medical University, Taichung, 40402 Taiwan

**Keywords:** Time-fractional Joseph–Egri equation, Exact soliton solutions, Jacobi elliptic function approach, Tanh–Coth method, Fractional-order nonlinear waves, Jumarie’s fractional derivative, Nonlinear wave propagation, Applied mathematics, Computational science

## Abstract

This study introduces new exact soliton solutions of the time-fractional Joseph–Egri equation by employing the Tanh–Coth and Jacobi Elliptic Function methods. Using Jumarie’s modified Riemann–Liouville derivative, a wide variety of soliton structures—such as periodic, bell-shaped, W-shaped, kink, and anti-bell-shaped waves—are obtained and expressed through hyperbolic, trigonometric, and Jacobi functions. The analysis reveals the significant impact of fractional-order derivatives on soliton dynamics, with graphical illustrations highlighting their physical relevance. This work expands the known solution space of the fractional Joseph–Egri equation, demonstrates the effectiveness of advanced analytical techniques, and provides fresh insights into the behavior of fractional nonlinear waves, with potential applications in physics and engineering.

## Introduction

Nonlinear partial differential equations (NLPDEs)^1–3^ are used to explain complex phenomena in a variety of fields, notably physics, engineering, mathematics and many other scientific fields. A variety of NLPDEs are used in fluid dynamics to describe shallow-water waves. These equations include the Bateman–Burgers equation, Boomeron equation, Boltzmann equation, Buckmaster equation, Chafee-Infante equation, Stochastic Zakharov–Kuznetsov equation, Radhakrishnan–Kundu–Lakshmanan (RKL) equation, Clairaut equation, Clarke’s equation, Dym equation, FitzHugh–Nagumo model, Davey–Stewartson, Kopel triopoly model^4^, (2+1)-dimensional complex modifed Korteweg-de-Vries equation^5^ and memristor cellular neural networks^6^. Determining the traveling-wave solutions of NLPDEs is the main physical issue. Several effective techniques have been presented for solving NLPDEs. These include the Sardar sub-equation method^7^, unified transform method^8^, the tanh-coth function method^9^, Hirota bilinear transformation^10^, tanh and Sardar techniques^11^, RPE method^12^, the TGD Techniques^13^, the Rational-expansion technique^14^, the P-expansion method^15^, and GERF method^16^, as well as the FFI method^17^, the FFV method^18^, the modified F-expansion method^19^, the Extended HB method^20^, the TVE method^21^, and variational methods^22^. Other notable studies that have been documented in the literature and embrace the nonlinearity of real-world problems are noteworthy.

As a result of L’Hopital’s inquiry to Leibniz regarding the $\frac{1}{2}$ derivative in a 1695 letter, fractional calculus originate. Afterwards, fractional, irrational, and complex derivatives were taken into account by researchers. Fractional-order derivatives can also be used to describe an extensive variety of physical phenomena, notably gravity, heat, elasticity, sound electrostatics, diffusion, and several others. The significance of the fractional-order derivative has led to the proposal of several definitions, such as the Davidson derivative^23^, Riemann–Liouville fractional^24^, conformable fractional^25^, Weyl derivative^26^, Atangana derivative^27^, conformable derivative^28,29^, Hadamard derivative^30^, Miller–Ross derivative^31^, Marchaud derivative^32^, Sonin–Letnikov derivative^33^, Erdélyi–Kober derivative^34^, Grünwald–Letnikov derivative^35^, and Katugampola derivative^36^.

Despite the fact that it is still unidirectional, the Joseph–Egri (JE) equation^37^ has many features in common with the Benjamin–Bona–Mahony equation as well as the KDV equation. For long waves with small amplitudes, the JE equation’s initial value problem was demonstrated to be well-posed by Bona and Chen in 1999. Furthermore, the solutions of this equation agree with those of the BBM equation and the KdV equation for small waves. But the JE equation is still not fully integrable, in contrast to the KdV equation. The traveling waves model of the Joseph–Egri equation has been extensively investigated as an essential framework for understanding the propagation of waves. The JE equation’s exact solutions were obtained by applying a variety of techniques, including the $(\frac{{{G^\prime }}}{G})$-expansion method^38^, extended HB method^39^, ESE method^40^, exp$\left( { - \phi \left( \xi \right) } \right)$-expansion method^41^, AE technique^42^, bifurcation method^43^, Cosine-function method^44^, and many others.

Fluids play a vital role in numerous scientific and engineering disciplines, from heat transfer to lubrication and fluid dynamics. However, the typical fluids such as water, oil, and ethylene glycol tend to be limited by their thermal conductivity, which inhibits their effectiveness in high-performance applications. In order to eliminate these constraints, scientists have introduced nanofluids—liquids with suspended nanoparticles that enhance thermal and rheological characteristics greatly. These nanoparticles, commonly composed of metals, oxides, or carbon materials, enhance heat transfer, stability, and overall efficiency. Nanofluids find broad applications in cooling systems, biomedical systems, and renewable energy systems because of their superior thermophysical properties. Their increased thermal conductivity, lowered viscosity, and increased stability make them a promising replacement for traditional fluids. Consequently, recent studies investigate their use in next-generation engineering applications such as microfluidics and heat exchangers.

In recent years, analytical methods like the Jacobi elliptic function (JEF) and Tanh–Coth techniques have been effectively used to obtain exact soliton solutions for nonlinear and fractional differential equations. The JEF method, for instance, has been applied to the improved mKdV equation with conformable derivatives^45^ and to equations involving truncated M-fractional derivatives^46,47^. Other works reported shock and periodic solutions for the fractional-time Gardner equation^48^, and bifurcation structures in generalized nonlinear Schrödinger equations^49^. Recent studies further enriched this field: Ref.^50^ explored diverse soliton structures and modulation instability analysis, Ref.^51^ investigated higher-order nonlinearities, and Ref.^52^ focused on optical models with complex nonlinear terms. Inspired by these advances, this study applies both the JEF and Tanh–Coth methods to the time-fractional Joseph–Egri (TFJE) equation to uncover new soliton solutions and better understand fractional wave propagation.

In addition to the techniques applied in this study, several recent works have demonstrated the effectiveness of related analytical methods in exploring complex nonlinear models. For instance, innovative soliton solutions were obtained for a (2+1)-dimensional generalized KdV equation using two effective approaches^53^, diverse soliton wave structures were analyzed for coupled nonlinear Schrödinger equations in birefringent fibers with higher nonlinearities by employing the modified extended mapping algorithm^54^, and the traveling and soliton waves of the extended (3+1)-dimensional Kadomtsev–Petviashvili equation in fluid were investigated^55^. Furthermore, invariant solitons and traveling-wave solutions for a higher-order nonlinear Schrödinger equation in an optical fiber were derived using an improved tanh-function algorithm^56^. These studies support the plausibility and relevance of applying the Jacobi elliptic function and Tanh-Coth methods in the current work.

In the present paper, A significant nonlinear partial differential equation in mathematical physics, named TFJE, is studied analytically. To address the fractional derivatives within the TFJE, we employ Jumarie’s fractional derivative, specifically the modified Riemann–Liouville (RL) definition^57^. Jumarie’s modified Riemann–Liouville (RL) derivative is chosen for its ability to preserve key properties of classical differentiation while effectively modeling memory effects in fractional systems. It simplifies the application of analytical methods like the Jacobi elliptic function and Tanh–Coth methods. This ensures consistency in deriving diverse soliton solutions for the TFJE equation. This approach allows us to derive analytical solutions for the TFJE equation, which is formulated as follows:

1 $$\begin{aligned} {\rho _t} + {\rho _x} + \varpi \rho {\rho _x} + {\rho _{x tt}}=0, \end{aligned}$$

which provides an essential framework for describing the propagation of waves, with $\varpi$ representing a nonzero constant.

The representation of the Joseph–Egri model with the modified RL fractional derivative is denoted as follows:

2 $$\begin{aligned} D_t^\varphi \rho + D_x^{\alpha } \rho + \varpi \rho D_x^{\alpha } \rho + D_x^{\alpha } D_t^{2\varphi }\rho =0 ,~x,t > 0 ,~~\varphi ,{\alpha }\in (0,1]. \end{aligned}$$

In Eq. (1), $\rho (x,t)$ denotes the wave profile or amplitude, $\rho _t = \frac{\partial \rho }{\partial t}$ is the time derivative, $\rho _x = \frac{\partial \rho }{\partial x}$ is the spatial derivative, $\rho _{x tt} = \frac{\partial ^3 \rho }{\partial x \partial t^2}$ is the mixed third-order derivative representing dispersive effects, and $\varpi$ is a nonzero constant characterizing the strength of nonlinearity. In the fractional form given by Eq. (2), $D_t^\varphi$ and $D_t^{2\varphi }$ are the modified Riemann–Liouville fractional derivatives of order $\varphi$ and $2\varphi$ with respect to time, while $D_x^\alpha$ is the modified Riemann–Liouville fractional derivative of order $\alpha$ with respect to space. These fractional derivatives incorporate memory and hereditary effects into the model, enhancing its ability to describe complex wave dynamics. The mathematical framework of the Joseph–Egri equation is initially presented in its classical form in Eq. (1), describing wave propagation with nonlinear and dispersive effects, where $\varpi$ is a nonzero constant governing the nonlinear interaction. To generalize this model for fractional-order dynamics, Equation (2) introduces the modified Riemann–Liouville (RL) fractional derivative, denoted as $D_t^\varphi$ and $D_x^\alpha$, which accounts for memory effects and anomalous diffusion in wave behavior. The term $D_x^\alpha D_t^{2\varphi } \rho$ extends the dispersive characteristics by incorporating fractional orders in both space and time. This fractional formulation enhances the model’s applicability to complex physical systems where traditional integer-order derivatives fail to capture long-range interactions and hereditary properties. The solutions to this extended equation are obtained using the Jacobi Elliptic Function and Tanh–Coth methods, demonstrating their capability to derive diverse soliton and periodic solutions. These methods are simple, straightforward, and applicable to higher-order NLPDEs, enabling the derivation of diverse soliton and periodic solutions. These approaches have never been applied to our model previously.

Despite extensive research on nonlinear fractional differential equations, the time-fractional Joseph–Egri equation remains insufficiently explored, particularly in understanding how fractional derivatives influence its complex soliton dynamics. Prior studies have largely addressed integer-order versions, leaving a critical gap in characterizing fractional-order effects on wave propagation. This work fills this gap by applying Jumarie’s modified Riemann–Liouville derivative alongside the Jacobi Elliptic Function and Tanh–Coth methods to derive new and diverse exact soliton solutions. The study’s novelty lies in significantly expanding the solution space of the fractional Joseph–Egri equation and providing deeper insights into fractional nonlinear wave phenomena relevant to modern physics and engineering applications.

The structure of this paper is organized as follows: Section introduces the definition and properties of fractional derivatives. In Section, we conduct a mathematical analysis of the TFJE equation. Section covers the foundational concepts of the Jacobi elliptic function and the tanh–coth methods. Section details the application of these analytical methods to the TFJE. Section discusses the results obtained, while Section highlights the novel contributions of this work. Finally, Section concludes the paper with a summary of findings and final remarks

## Theoretical framework

The following subsection covers the definitions and fundamental attributes of fractional derivative.

### Modified Riemann–Liouville derivative by Jumarie

#### Definition

For an order of $\varphi$, the Jumarie’s modified RL derivative^57^ is defined as

$$\begin{aligned} D_t^\varphi \rho (t) = \left\{ {\begin{array}{*{20}{c}} {{\textstyle {1 \over {\Gamma (1 - \varphi )}}}\int \limits _0^t {{{(t -\wp )}^{ - \varphi - 1}}(\rho (\wp ) - \rho (0))d\wp ,\hspace{0.75in}\varphi< 0,} }\\ {{\textstyle {1 \over {\Gamma (1 - \varphi )}}}\frac{d}{{dt}}\int \limits _0^t {{{(t - \wp )}^{ - \varphi }}(\rho (\wp ) - \rho (0))d\wp ,\hspace{0.5in}0< \varphi< 1,} }\\ {\,\quad {\hspace{1.0pt}} {{({\rho ^{(n)}}(t))}^{\varphi - n}},\hspace{1.15in}n \le \varphi < n + 1,n \ge 1}, \end{array}} \right. \end{aligned}$$

where *n* is an integer, just less than the real number $\varphi$. These are some of the characteristics of the Jumarie’s modified RL derivative.

1. $$D_t^\varphi {\Psi ^N} = {\textstyle {{\Gamma (N + 1)} \over {\Gamma (N + 1 - \varphi )}}}{\Psi ^{N - \varphi }},N > 0$$.
2. $$D_t^\varphi \ell = 0,\;\;\;\;\;\ell$$ is a constant.
3. $D_t^\varphi ({\ell _1}s(t) + {\ell _2}r(t)) = {\ell _1}D_t^\varphi s(t) + {\ell _2}D_t^\varphi r(t)$, ${\ell _1}$ and ${\ell _2}$ are constants.
4. $$D_t^\varphi (s(t)r(t)) = r(t)D_t^\varphi s(t) + s(t)D_t^\varphi r(t)$$.
5. $$D_t^\varphi s(r(t)) = s_r^\prime [s(t)]D_t^\varphi r(t) = D_t^\varphi s[r(t)]{({r^\prime }(t))^\varphi }$$.

## Analytical procedure assessment

This section outlines the analytical procedure used to derive the wave equation for the time-fractional Joseph–Egri model. By applying a wave transformation and substituting it into the fractional equation, we simplify the equation and integrate to obtain a solvable expression for the wave function $\rho$. The wave equation for the TFJE can be derived using the following wave transformation, as shown in Eq. (1).

3 $$\begin{aligned} \rho (x ,t) =\rho (\wp ),\;\; \wp =\frac{{{\hbar _1}{x ^\alpha }}}{{\Gamma (1 + \alpha )}} - \frac{{{\hbar _2}{t^\varphi }}}{{\Gamma (1 + \varphi )}}, \end{aligned}$$

has been put to use. Whereas $\hbar _{1}$ and $\hbar _{2}$ are nonzero arbitrary parameters. By using the transformations of Eq. (3) in Eq. (2), yields

4 $$\begin{aligned} ({\hbar _1} - {\hbar _2})\rho ^\prime + \varpi {\hbar _1}\rho \rho ^{\prime ^2}\rho ^{\prime \prime \prime } = 0, \end{aligned}$$

where ${\rho ^\prime }=\frac{{d\rho }}{{d\wp }}$. By setting the integration constant to zero, Eq. (4) can be integrated once, resulting in:

5 $$\begin{aligned} ({\hbar _1} - {\hbar _2})\rho + \varpi {\hbar _1}\frac{{{\rho ^2}}}{2} + {\hbar _1}\hbar _2^2\rho ^{\prime \prime } = 0. \end{aligned}$$

## Algorithms

### Jacobi elliptic function method

In this section, we outline the process for solving NLPDEs using the JEF method^58^.

- *Step 1* Given two independent variables, *x* and *t*, the following partial differential equation represents a nonlinear system:
  6 $$\begin{aligned} U\left( \rho , \rho _t, \rho _x, \rho _{tt}, \rho _{xx}, \rho _{xt}, \ldots \right) = 0, \end{aligned}$$
  where *U* is a polynomial involving the function $\rho (x, t)$, its partial derivatives, and includes the highest order derivatives and nonlinear terms. The unknown function $\rho (x, t)$ is what we aim to solve. The primary steps for this procedure are detailed below.
- *Step 2* We introduce a transformation that combines the independent variables *x* and *t* into a single variable $\wp = x \pm \mu t$, and assume that:
  7 $$\begin{aligned} \rho (x, t) = \rho (\wp ), \quad \wp = x \pm \mu t. \end{aligned}$$
  Using the transformation in Eq. (7), the original PDE (6) is reduced to an ordinary differential equation (ODE):
  8 $$\begin{aligned} U\left( \rho , \rho ', \rho '', \ldots \right) = 0, \end{aligned}$$
  where $\rho '(\wp ) = \frac{d\rho }{d\wp }$, and *U* is now a polynomial in $\rho (\wp )$ and its derivatives.
- *Step 3* We propose that the solution to Eq. (8) can be expressed as:
  9 $$\begin{aligned} \rho (\wp ) = \sum _{p=0}^R g_p \left[ \mho (\wp )\right] ^p, \quad g_p \ne 0, \end{aligned}$$
  where $\mho (\wp ) = \text {sn}(\wp , q)$ with $0< q < 1$, and the constants $g_p$ are to be determined.
- *Step 4* To determine the positive integer *R*, we balance the highest order derivatives with the nonlinear terms in either Eqs. (6) or (8). Furthermore, we define the degree of $\rho (\wp )$ as $D(\rho (\wp )) = R$. This characterization assists in determining the degrees of other related expressions:
  10 $$\begin{aligned} D\left( \frac{d^n \rho }{d\wp ^n}\right) = R + n, \quad D\left( \rho ^p \left( \frac{d^n \rho }{d\wp ^n}\right) ^s\right) = Rp + s(R + n). \end{aligned}$$
  This allows us to establish the value of *R* using Eq. (10).
- *Step 5* By substituting Eq. (9) into Eq. (8) and setting the coefficients of $\mho (\wp )^p$ to zero, we generate a system of algebraic equations. Solving this system yields the values of $g_p$. Finally, the exact solutions to Eq. (6) are obtained by substituting the calculated $g_p$ values back into Eq. (9).

### Algorithm of Jacobi elliptic function method

- Take a nonlinear differential equation.
- Use a traveling wave transformation.
- Assume a solution in terms of Jacobi elliptic functions.
- Find the highest power in the assumed solution.
- Substitute the assumed solution into the transformed equation.
- Employ Jacobi elliptic function identities.
- Solve for the unknown parameters.
- Analyze special cases.
- Obtained the required solution.

### Tanh–Coth method

In this section, we explore solving an NLPDE using the Tanh–Coth method^59^. The general form of the NLPDE is presented as follows:

*Step 1*

11 $$\begin{aligned} F(\aleph ,\;{\aleph _t},\;{\aleph _x},\;{\aleph _{tt}},\;{\aleph _{xx}},\;{\aleph _{xt}},\ldots ) = 0, \end{aligned}$$

where $\aleph (x, t) = \aleph (\wp )$ is an unknown function, and *F* represents a polynomial in $\aleph$. We apply the wave transformation:

12 $$\begin{aligned} \wp = \eta x + \mu t, \end{aligned}$$

where $\eta$ and $\mu$ are nonzero real constants. This transformation converts Eq. (11) into the following ODE:

13 $$\begin{aligned} F(\aleph ,\;{\aleph '},\;{\aleph ''},\ldots ) = 0, \end{aligned}$$

where *F* is a polynomial in $\aleph$, and the ordinary derivatives of $\aleph$ with respect to $\wp$ are denoted by the primes.

*Step 2* Suppose that, the nonlinear partial differential Eq. (11) admits the following solution:

14 $$\begin{aligned} \aleph (\wp ) = \sum \limits _{p = 0}^R {k_p \left[ \chi (\wp )\right] ^p}, \quad k_p \ne 0, \end{aligned}$$

where $k_p$ are real constants to be determined, and $\chi (\wp )$ satisfies the ODE given by:

15 $$\begin{aligned} \chi (\wp ) = \tanh (\wp ), \quad \text {or} \quad \chi (\wp ) = \coth (\wp ). \end{aligned}$$

*Step 3* We determine the value of *R* using the homogeneous balance method. After determining *R*, Eq. (13) is substituted by Eqs. (14) and (15). Then, we equate the coefficients of each power of $\chi (\wp )$ to zero.

*Step 4* By solving for the required parameters, the given equation can be accurately addressed using this system of equations.

### Algorithm of tanh–coth method

- Take a nonlinear differential equation.
- Use a traveling wave transformation.
- Assume a solution in terms of hyperbolic functions.
- Find the greatest power in the hypothetical solution.
- Apply the assumed solution to the simplified equation.
- Apply hyperbolic function identities.
- Solve for the unknown parameters.
- Analyze special cases.
- Obtained the required solution.

## Application of mathematical methods

In this section, the Jacobi elliptic function method and the Tanh–Coth method are applied to obtain exact solutions of the time-fractional Joseph–Egri equation. These methods enable the transformation of the fractional partial differential equation into ordinary differential equations, facilitating analytical solution construction. While the Jacobi elliptic function method is particularly effective in generating periodic and multi-peak soliton solutions, the Tanh–Coth method excels at producing localized solitary waveforms such as kink and bell-shaped solitons. The complementary nature of these approaches allows for a comprehensive exploration of diverse wave structures, highlighting the rich dynamics captured by the fractional model.

### Jacobi elliptic function method

According to the JEF approach, $\rho \left( \wp \right)$ can be expressed as a finite series of JEF^60^, $\mho \left( \wp \right) = sn \left( {\wp ,q} \right)$ for $0< q < 1$, i.e

16 $$\begin{aligned} \rho (\wp ) = \sum \limits _{p = 0}^R {{g_p}{{\left[ {\mho (\wp )} \right] }^p}} ,\;\;\;{g_p}\;\ne \;0. \end{aligned}$$

By using the homogenous balancing principle between the terms ${\rho ^{\prime \prime }}$ and ${\rho ^2}$ of Eq. (5), gives

$$\begin{aligned} R+2=2R, \end{aligned}$$

so that

$$\begin{aligned} R=2. \end{aligned}$$

Thus Eq. (16) can be expressed as,

17 $$\begin{aligned} \rho (\wp ) = {g_0} + {g_1}\mho (\wp ) + {g_2}{\left( {\mho (\wp )} \right) ^2}, \end{aligned}$$

where ${g_0},\;{g_1}$ and ${g_2}$ are arbitrary constants which can be found later. The Equation (17) can be inserted into Eq. (5), yields

$$\begin{aligned} & {\hbar _1}{g_0} - {\hbar _2}{g_0} + \frac{1}{2}{\hbar _1}\varpi g_0^2 + {\hbar _1} \mho \left( \wp \right) {g_1} - {\hbar _2} \mho \left( \wp \right) {g_1} - {\hbar _1}\hbar _2^2 \mho \left( \wp \right) {g_1} -{\hbar _1}\hbar _2^2{q^2}\mho \left( \wp \right) {g_1} \\ & \quad + 2{\hbar _1}\hbar _2^2{q^2}\mho {\left( \wp \right) ^3}{g_1} + {\hbar _1}\varpi \mho \left( \wp \right) {g_0}{g_1} + \frac{1}{2}{\hbar _1}\varpi \mho {\left( \wp \right) ^2}g_1^2 + 2{\hbar _1}\hbar _2^2{g_2} + {\hbar _1}\mho {\left( \wp \right) ^2}{g_2} - {\hbar _2}\mho {\left( \wp \right) ^2}{g_2} \\ & \quad - 4{\hbar _1}\hbar _2^2\mho {\left( \wp \right) ^2}{g_2} - 4{\hbar _1}\hbar _2^2{q^2}\mho {\left( \wp \right) ^2}{g_2} + 6{\hbar _1}\hbar _2^2{q^2}\mho {\left( \wp \right) ^4}{g_2} + {\hbar _1}\varpi \mho {\left( \wp \right) ^2}{g_0}{g_2} + {\hbar _1}\varpi \mho {\left( \wp \right) ^3}{g_1}{g_2} \\ & \quad +\frac{1}{2}{\hbar _1}\varpi \mho {\left( \wp \right) ^4}g_2^2=0. \end{aligned}$$

When $p = 0, 1, 2, 3, 4$, and all of the coefficients of $\mho (\wp )^p$ are set to zero, the following occurs:

18 $$\begin{aligned} \begin{aligned} \mho {\left( \wp \right) ^0}:{\hbar _1}{g_0} - {\hbar _2}{g_0} + \frac{1}{2}{\hbar _1}\varpi g_0^2 + 2{\hbar _1}\hbar _2^2{g_2}=0,\\ \mho {\left( \wp \right) ^1}:{\hbar _1}{g_1} - {\hbar _2}{g_1} - {\hbar _1}\hbar _2^2{g_1} - {\hbar _1}\hbar _2^2{q^2}{g_1} + {\hbar _1}\varpi {g_0}{g_1}=0,\\ \mho {\left( \wp \right) ^2}: \frac{1}{2}{\hbar _1}\varpi g_1^2 + {\hbar _1}{g_2} - {\hbar _2}{g_2} - 4{\hbar _1}\hbar _2^2{g_2} - 4{\hbar _1}\hbar _2^2{q^2}{g_2} + {\hbar _1}\varpi {g_0}{g_2}=0,\\ \mho {\left( \wp \right) ^3}: 2{\hbar _1}\hbar _2^2{q^2}{g_1} + {\hbar _1}\varpi {g_1}{g_2}=0,\\ \mho {\left( \wp \right) ^4}: 6{\hbar _1}\hbar _2^2{q^2}{g_2} + \frac{1}{2}{\hbar _1}\varpi g_2^2=0. \end{aligned} \end{aligned}$$

When the aforementioned Equations are solved for ${g_0},\;{g_1},\;{g_2},\; and\;{\hbar _1}$, we get

*Case 1*

If $${g_0} = \frac{{ - {\hbar _1} + {\hbar _2} + 4{\hbar _1}{\hbar _2}^2 + 4{\hbar _1}{\hbar _2}^2{q^2}}}{{{\hbar _1}\varpi }}$$, $${g_1} = 0$$, $${g_2} = \frac{{{\hbar _1}^2 - 2{\hbar _1}{\hbar _2} + {\hbar _2}^2 - 16{\hbar _1}^2{\hbar _2}^4 - 32{\hbar _1}^2{\hbar _2}^4{q^2} - 16{\hbar _1}^2{\hbar _2}^4{q^4}}}{{4{\hbar _1}^2\varpi {\hbar _2}^2}}$$,

then the periodic solution of Eq. (17) is

19 $$\begin{aligned} \begin{aligned} \rho _{1,1}(x,t) = \frac{{{\hbar _1}^2 - 2{\hbar _1}{\hbar _2} + {\hbar _2}^2 - 16{\hbar _1}^2{\hbar _2}^4 - 32{\hbar _1}^2{\hbar _2}^4{q^2} - 16{\hbar _1}^2{\hbar _2}^4{q^4}}}{{4{\hbar _1}^2\varpi {\hbar _2}^2}}{\left( {sn\left( {\wp ,q} \right) } \right) ^2}\\ +\frac{{ - {\hbar _1} + {\hbar _2}+ 4{\hbar _1}{\hbar _2}^2 + 4{\hbar _1}{\hbar _2}^2{q^2}}}{{{\hbar _1}\varpi }}. \end{aligned} \end{aligned}$$

If $q \rightarrow 1$, then Eq. (19) becomes

20 $$\begin{aligned} \rho _{1,2}(x,t) = \frac{{ - {\hbar _1} + {\hbar _2} + 8{\hbar _1}{\hbar _2}^2}}{{{\hbar _1}\varpi }} + \frac{{{\hbar _1}^2 - 2{\hbar _1}{\hbar _2} + {\hbar _2}^2 - 64{\hbar _1}^2{\hbar _2}^4}}{{4{\hbar _1}^2\varpi {\hbar _2}^2}}{\left( {\tanh \wp } \right) ^2}. \end{aligned}$$

*Case 2*

If ${g_0} = \frac{{ - 2{\hbar _1}\varpi + 2\varpi {\hbar _2} - \sqrt{{{\left( { - 2{\hbar _1}\varpi + 2\varpi {\hbar _2}} \right) }^2} + 192{\hbar _1}^2{\varpi ^2}{\hbar _2}^4{q^2}} }}{{2{\hbar _1}{\varpi ^2}}}$, ${g_1} = 0$, ${g_2} = - \frac{{12{\hbar _2}^2{q^2}}}{\varpi }$, then the

periodic solution of Eq. (17) is

21 $$\begin{aligned} \begin{aligned} \rho _{2,1}(x,t) = \frac{{ - 2{\hbar _1}\varpi + 2\varpi {\hbar _2} - \sqrt{{{\left( { - 2{\hbar _1}\varpi + 2\varpi {\hbar _2}} \right) }^2} + 192{\hbar _1}^2{\varpi ^2}{\hbar _2}^4{q^2}} }}{{2{\hbar _1}{\varpi ^2}}} \\ - \frac{{12{\hbar _2}^2{q^2}}}{\varpi }{\left( {sn\left( {\wp ,q} \right) } \right) ^2}. \end{aligned} \end{aligned}$$

If $q \rightarrow 1$, then Eq. (21) becomes

$$\begin{aligned} \begin{aligned} \rho _{2,2}(x,t) = \frac{{ - 2{\hbar _1}\varpi + 2\varpi {\hbar _2} - \sqrt{{{\left( { - 2{\hbar _1}\varpi + 2\varpi {\hbar _2}} \right) }^2} + 192{\hbar _1}^2{\varpi ^2}{\hbar _2}^4} }}{{2{\hbar _1}{\varpi ^2}}} \\ - \frac{{12{\hbar _2}^2}}{\varpi }{\left( {\tanh \wp } \right) ^2}, \end{aligned} \end{aligned}$$

*Case 3*

If ${g_0} = \frac{{ - 2{\hbar _1}\varpi + 2\varpi {\hbar _2} + \sqrt{{{\left( { - 2{\hbar _1}\varpi + 2\varpi {\hbar _2}} \right) }^2} + 192{\hbar _1}^2{\varpi ^2}{\hbar _2}^4{q^2}} }}{{2{\hbar _1}{\varpi ^2}}}$, ${g_1} = 0$, ${g_2} = - \frac{{12{\hbar _2}^2{q^2}}}{\varpi }$, then the

periodic solution of Eq. (17) is

22 $$\begin{aligned} \begin{aligned} \rho _{3,1}(x,t) = \frac{{ - 2{\hbar _1}\varpi + 2\varpi {\hbar _2} + \sqrt{{{\left( { - 2{\hbar _1}\varpi + 2\varpi {\hbar _2}} \right) }^2} + 192{\hbar _1}^2{\varpi ^2}{\hbar _2}^4{q^2}} }}{{2{\hbar _1}{\varpi ^2}}}\\frac{{12{\hbar _2}^2{q^2}}}{\varpi }{\left( {sn\left( {\wp ,q} \right) } \right) ^2}. \end{aligned} \end{aligned}$$

If $q \rightarrow 1$, then Eq. (22) becomes

$$\begin{aligned} \begin{aligned} \rho _{3,2}(x,t) = \frac{{ - 2{\hbar _1}\varpi + 2\varpi {\hbar _2} + \sqrt{{{\left( { - 2{\hbar _1}\varpi + 2\varpi {\hbar _2}} \right) }^2} + 192{\hbar _1}^2{\varpi ^2}{\hbar _2}^4{q^2}} }}{{2{\hbar _1}{\varpi ^2}}} \\ - \frac{{12{\hbar _2}^2{q^2}}}{\varpi }{\left( {\tanh \wp } \right) ^2}, \end{aligned} \end{aligned}$$

*Case 4*

If ${g_0} = \frac{{ - {\hbar _1} + {\hbar _2} + 4{\hbar _1}{\hbar _2}^2 + 4{\hbar _1}{\hbar _2}^2{q^2}}}{{{\hbar _1}\varpi }}$, ${g_1} = 0$, ${g_2} = - \frac{{12{\hbar _2}^2{q^2}}}{\varpi }$, then the periodic solution

of Eq. (17) is

23 $$\begin{aligned} \rho _{4,1}(x ,t) = \frac{{ - {\hbar _1} + {\hbar _2} + 4{\hbar _1}{\hbar _2}^2 + 4{\hbar _1}{\hbar _2}^2{q^2}}}{{{\hbar _1}\varpi }} - \frac{{12{\hbar _2}^2{q^2}}}{\varpi }{\left( {sn (\wp ,q)} \right) ^2}. \end{aligned}$$

If $q \rightarrow 1$, then Eq. (23) becomes

$$\begin{aligned} \rho _{4,2}(x ,t) = \frac{{ - {\hbar _1} + {\hbar _2} + 4{\hbar _1}{\hbar _2}^2 + 4{\hbar _1}{\hbar _2}^2{q^2}}}{{{\hbar _1}\varpi }} - \frac{{12{\hbar _2}^2{q^2}}}{\varpi }{\left( {\tanh (\wp )} \right) ^2}, \end{aligned}$$

*Case 5*

If ${g_0} = \frac{{ - {\hbar _1} + {\hbar _2} + {\hbar _1}{\hbar _2}^2 + {\hbar _1}{\hbar _2}^2{q^2}}}{{{\hbar _1}\varpi }}$, ${g_1} = - \frac{{2\mathrm{{i}}\sqrt{3} {\hbar _2}^2\sqrt{{q^2} + {q^4}} }}{\varpi }$, ${g_2} = - \frac{{2{\hbar _2}^2{q^2}}}{\varpi }$, then the periodic

solution of Eq. (17) is

24 $$\begin{aligned} \begin{aligned} \rho _{5,1}(x ,t) = \frac{{ - {\hbar _1} + {\hbar _2} + {\hbar _1}{\hbar _2}^2 + {\hbar _1}{\hbar _2}^2{q^2}}}{{{\hbar _1}\varpi }} - \frac{{2\mathrm{{i}}\sqrt{3} {\hbar _2}^2\sqrt{{q^2} + {q^4}} }}{\varpi }\left( {sn(\wp ,q)} \right) \\ - \frac{{2{\hbar _2}^2{q^2}}}{\varpi }{\left( {sn(\wp ,q)} \right) ^2}. \end{aligned} \end{aligned}$$

If $q \rightarrow 1$, then Eq. (24) becomes

$$\begin{aligned} \begin{aligned} \rho _{5,2}(x ,t) = \frac{{ - {\hbar _1} + {\hbar _2} + {\hbar _1}{\hbar _2}^2 + {\hbar _1}{\hbar _2}^2{q^2}}}{{{\hbar _1}\varpi }} - \frac{{2\mathrm{{i}}\sqrt{3} {\hbar _2}^2\sqrt{{q^2} + {q^4}} }}{\varpi }\left( {\tanh (\wp )} \right) \\ - \frac{{2{\hbar _2}^2{q^2}}}{\varpi }{\left( {\tanh (\wp )} \right) ^2}. \end{aligned} \end{aligned}$$

*Case 6*

If ${g_0} = \frac{{ - {\hbar _1} + {\hbar _2} + {\hbar _1}{\hbar _2}^2 + {\hbar _1}{\hbar _2}^2{q^2}}}{{{\hbar _1}\varpi }}$, ${g_1} = \frac{{2\mathrm{{i}}\sqrt{3} {\hbar _2}^2\sqrt{{q^2} + {q^4}} }}{\varpi }$, ${g_2} = - \frac{{2{\hbar _2}^2{q^2}}}{\varpi }$, then the periodic

solution of Eq. (17) is

25 $$\begin{aligned} \begin{aligned} \rho _{6,1}(x ,t) = \frac{{ - {\hbar _1} + {\hbar _2} + {\hbar _1}{\hbar _2}^2 + {\hbar _1}{\hbar _2}^2{q^2}}}{{{\hbar _1}\varpi }} + \frac{{2\mathrm{{i}}\sqrt{3} {\hbar _2}^2\sqrt{{q^2} + {q^4}} }}{\varpi }\left( {sn(\wp ,q)} \right) \\ - \frac{{2{\hbar _2}^2{q^2}}}{\varpi }{\left( {sn(\wp ,q)} \right) ^2}. \end{aligned} \end{aligned}$$

If $q \rightarrow 1$, then Eq. (25) becomes

$$\begin{aligned} \begin{aligned} \rho _{6,2}(x ,t) = \frac{{ - {\hbar _1} + {\hbar _2} + {\hbar _1}{\hbar _2}^2 + {\hbar _1}{\hbar _2}^2{q^2}}}{{{\hbar _1}\varpi }} + \frac{{2\mathrm{{i}}\sqrt{3} {\hbar _2}^2\sqrt{{q^2} + {q^4}} }}{\varpi }\left( {\tanh (\wp )} \right) \\ - \frac{{2{\hbar _2}^2{q^2}}}{\varpi }{\left( {\tanh (\wp )} \right) ^2}. \end{aligned} \end{aligned}$$

*Case 7*

If ${g_0} = \frac{{ - {\hbar _1} + {\hbar _2} + {\hbar _1}{\hbar _2}^2 + {\hbar _1}{\hbar _2}^2{q^2}}}{{{\hbar _1}\varpi }}$, ${g_1} = - \frac{{6\mathrm{{i}}\sqrt{2} {\hbar _2}^2\sqrt{{q^2} + {q^4}} }}{\varpi }$, ${g_2} = - \frac{{12{\hbar _2}^2{q^2}}}{\varpi }$, then the periodic

solution of Eq. (17) is

26 $$\begin{aligned} \begin{aligned} \rho _{7,1}(x ,t) = \frac{{ - {\hbar _1} + {\hbar _2} + {\hbar _1}{\hbar _2}^2 + {\hbar _1}{\hbar _2}^2{q^2}}}{{{\hbar _1}\varpi }} - \frac{{6\mathrm{{i}}\sqrt{2} {\hbar _2}^2\sqrt{{q^2} + {q^4}} }}{\varpi }\left( {sn (\wp ,q)} \right) \\ - \frac{{12{\hbar _2}^2{q^2}}}{\varpi }{\left( {sn (\wp ,q)} \right) ^2}. \end{aligned} \end{aligned}$$

If $q \rightarrow 1$, then Eq. (26) becomes

$$\begin{aligned} \begin{aligned} \rho _{7,2}(x ,t) = \frac{{ - {\hbar _1} + {\hbar _2} + {\hbar _1}{\hbar _2}^2 + {\hbar _1}{\hbar _2}^2{q^2}}}{{{\hbar _1}\varpi }} - \frac{{6\mathrm{{i}}\sqrt{2} {\hbar _2}^2\sqrt{{q^2} + {q^4}} }}{\varpi }\left( {\tanh (\wp )} \right) \\ - \frac{{12{\hbar _2}^2{q^2}}}{\varpi }{\left( {\tanh (\wp )} \right) ^2}, \end{aligned} \end{aligned}$$

*Case 8*

If ${g_0} = \frac{{ - {\hbar _1} + {\hbar _2} + {\hbar _1}{\hbar _2}^2 + {\hbar _1}{\hbar _2}^2{q^2}}}{{{\hbar _1}\varpi }}$, ${g_1} = \frac{{6\mathrm{{i}}\sqrt{2} {\hbar _2}^2\sqrt{{q^2} + {q^4}} }}{\varpi }$, ${g_2} = - \frac{{12{\hbar _2}^2{q^2}}}{\varpi }$, then the periodic

solution of Eq. (17) is

27 $$\begin{aligned} \begin{aligned} \rho _{8,1}(x,t) = \frac{{ - {\hbar _1} + {\hbar _2} + {\hbar _1}{\hbar _2}^2 + {\hbar _1}{\hbar _2}^2{q^2}}}{{{\hbar _1}\varpi }} + \frac{{6\mathrm{{i}}\sqrt{2} {\hbar _2}^2\sqrt{{q^2} + {q^4}} }}{\varpi }\left( {sn (\wp ,q)} \right) \\ - \frac{{12{\hbar _2}^2{q^2}}}{\varpi }{\left( {sn (\wp ,q)} \right) ^2}. \end{aligned} \end{aligned}$$

If $q \rightarrow 1$, then Eq. (27) becomes

$$\begin{aligned} \begin{aligned} \rho _{8,2}(x ,t) = \frac{{ - {\hbar _1} + {\hbar _2} + {\hbar _1}{\hbar _2}^2 + {\hbar _1}{\hbar _2}^2{q^2}}}{{{\hbar _1}\varpi }} + \frac{{6\mathrm{{i}}\sqrt{2} {\hbar _2}^2\sqrt{{q^2} + {q^4}} }}{\varpi }\left( {\tanh (\wp )} \right) \\ - \frac{{12{\hbar _2}^2{q^2}}}{\varpi }{\left( {\tanh (\wp )} \right) ^2}, \end{aligned} \end{aligned}$$

where $\hbar _{1}$, $\hbar _{2}\;and \;\varpi$ are the arbitrary parameters.

Similarly, we can substitute $sn\left( {\wp ,q} \right)$ with $cn\left( {\wp ,q} \right)$ and $dn\left( {\wp ,q} \right)$ to find unique solutions of Eq. (5).

### Tanh–Coth method

The TC method is currently employed^61^. Assuming the following solution for the NLPDE in Eq. (5):

28 $$\begin{aligned} \aleph \left( \wp \right) = {\sum \limits _{p = 0}^R {{k_p}\left[ {\chi \left( \wp \right) } \right] } ^p} ,\;\;\;{k_p}\;\ne \;0, \end{aligned}$$

where $\chi (\wp )=\tanh \wp$ ( or $\chi (\wp )=\coth \wp$) and $k_{p}$ are real constants. To determine the parameter *R*, we balance ${\rho ^{\prime \prime }}$ with ${\rho ^2}$ in Eq. (5), which yields $R=2$. So Eq. (28) can be written as,

29 $$\begin{aligned} \aleph (\wp ) = {k_0} + {k_1}\chi \left( \wp \right) + {k_2}\chi {\left( \wp \right) ^2}, \end{aligned}$$

where ${k_0}\;{k_1}$ and ${k_2}$ are arbitrary constants which can be found later. The Eq. (29) is substituted into Eq. (5), yields

30 $$\begin{aligned} \begin{aligned} {\hbar _1}{k_0} - {\hbar _2}{k_0} + \frac{1}{2}{\hbar _1}\varpi k_0^2 + 2{\hbar _1}\hbar _2^2{k_2} + {\hbar _1}{k_1}\chi \left( \wp \right) - {\hbar _2}{k_1}\chi \left( \wp \right) - 2{\hbar _1}\hbar _2^2{k_1}\chi \left( \wp \right) \\ + {\hbar _1}\varpi {k_0}{k_1}\chi \left( \wp \right) + \frac{1}{2}{\hbar _1}\varpi k_1^2\chi {\left( \wp \right) ^2} + {\hbar _1}{k_2}\chi {\left( \wp \right) ^2} - {\hbar _2}{k_2}\chi {\left( \wp \right) ^2} - 8{\hbar _1}\hbar _2^2{k_2}\chi {\left( \wp \right) ^2} \\ + {\hbar _1}\varpi {k_0}{k_2}\chi {\left( \wp \right) ^2} + 2{\hbar _1}\hbar _2^2{k_1}\chi {\left( \wp \right) ^3} + {\hbar _1}\varpi {k_1}{k_2}\chi {\left( \wp \right) ^3} + 6{\hbar _1}\hbar _2^2{k_2}\chi {\left( \wp \right) ^4} \\ + \frac{1}{2}{\hbar _1}\varpi k_2^2\chi {\left( \wp \right) ^4} = 0. \end{aligned} \end{aligned}$$

By setting each coefficient of $\chi (\wp )^p$ to zero, we obtain the following system of algebraic equations:

$$\begin{aligned} \begin{aligned} \chi {\left( \wp \right) ^0}:{\hbar _1}{k_0} - {\hbar _2}{k_0} + \frac{1}{2}{\hbar _1}\varpi k_0^2 + 2{\hbar _1}{\hbar _2}^2{k_2}=0,\\ \chi {\left( \wp \right) ^1}:{\hbar _1}{k_1} - {\hbar _2}{k_1} - 2{\hbar _1}{\hbar _2}^2{k_1} + {\hbar _1}\varpi {k_0}{k_1}=0,\\ \chi {\left( \wp \right) ^2}:\frac{1}{2}{\hbar _1}\varpi k_1^2 + {\hbar _1}{k_2} - {\hbar _2}{k_2} - 8{\hbar _1}{\hbar _2}^2{k_2} + {\hbar _1}\varpi {k_0}{k_2}=0,\\ \chi {\left( \wp \right) ^3}:2{\hbar _1}{\hbar _2}^2{k_1} + {\hbar _1}\varpi {k_1}{k_2}=0,\\ \chi {\left( \wp \right) ^4}:6{\hbar _1}{\hbar _2}^2{k_2} + \frac{1}{2}{\hbar _1}\varpi k_2^2=0.\\ \end{aligned} \end{aligned}$$

The solutions to the aforementioned equations yield the following families.

*Set 1* If ${k_0} = \frac{{ - {\hbar _1} + {\hbar _2} + 8{\hbar _1}{\hbar _2}^2}}{{\hbar _{1}\varpi }}$, ${k_1} = 0$ and ${k_2} = \frac{{{\hbar _1}^2 - 2{\hbar _1}{\hbar _2} + {\hbar _2}^2 - 64{\hbar _1}^2{\hbar _2}^4}}{{4{\hbar _1}^2\varpi {\hbar _2}^2}}$, then the following solution of Eq. (29) is obtained:

31 $$\begin{aligned} \aleph _{1,1}(x,t) = \frac{{ - {\hbar _1} + {\hbar _2} + 8{\hbar _1}{\hbar _2}^2}}{{\hbar _{1}\varpi }} +\frac{{{\hbar _1}^2 - 2{\hbar _1}{\hbar _2} + {\hbar _2}^2 - 64{\hbar _1}^2{\hbar _2}^4}}{{4{\hbar _1}^2\varpi {\hbar _2}^2}}{\left( {\tanh (\wp )} \right) ^2}, \end{aligned}$$

32 $$\begin{aligned} \aleph _{1,2}(x,t) = \frac{{ - {\hbar _1} + {\hbar _2} + 8{\hbar _1}{\hbar _2}^2}}{{\hbar _{1}\varpi }} + \frac{{{\hbar _1}^2 - 2{\hbar _1}{\hbar _2} + {\hbar _2}^2 - 64{\hbar _1}^2{\hbar _2}^4}}{{4{\hbar _1}^2\varpi {\hbar _2}^2}}{\left( {\coth (\wp )} \right) ^2}. \end{aligned}$$

*Set 2* If ${k_0} = \frac{{\left( {2 + 2\mathrm{{i}}} \right) {\hbar _2}^2}}{\varpi }$, ${k_1} = - \frac{{2\mathrm{{i}}\sqrt{6} {\hbar _2}^2}}{\varpi }$, ${k_2} = - \frac{{2{\hbar _2}^2}}{\varpi },$ and ${\hbar _1} = - \frac{{\mathrm{{i}}{\hbar _2}}}{{ - \mathrm{{i}} + 2{\hbar _2}^2}}$, then the following solution of Eq. (29) is obtained:

33 $$\begin{aligned} \aleph _{2,1}(x ,t) = \frac{{\left( {2 + 2\mathrm{{i}}} \right) {\hbar _2}^2}}{\varpi } - \frac{{2\mathrm{{i}}\sqrt{6} {\hbar _2}^2}}{\varpi }\tanh \left( \wp \right) - \frac{{2{\hbar _2}^2}}{\varpi }{\left( {\tanh (\wp )} \right) ^2}, \end{aligned}$$

34 $$\begin{aligned} \aleph _{2,2}(x ,t) = \frac{{\left( {2 + 2\mathrm{{i}}} \right) {\hbar _2}^2}}{\varpi } - \frac{{2\mathrm{{i}}\sqrt{6} {\hbar _2}^2}}{\varpi }\coth \left( \wp \right) - \frac{{2{\hbar _2}^2}}{\varpi }{\left( {\coth (\wp )} \right) ^2}. \end{aligned}$$

*Set 3* If ${k_0} = \frac{{4{\hbar _2}^2}}{\varpi }$, ${k_1} = 0$, ${k_2} = - \frac{{12{\hbar _2}^2}}{\varpi }$ and ${\hbar _1} = - \frac{{{\hbar _2}}}{{ - 1 + 4{\hbar _2}^2}}$, then the following solution of Eq. (29) is obtained:

35 $$\begin{aligned} \aleph _{3,1}(x ,t) = \frac{{4{\hbar _2}^2}}{\varpi } - \frac{{12{\hbar _2}^2}}{\varpi }{\left( {\tanh (\wp )} \right) ^2}, \end{aligned}$$

36 $$\begin{aligned} \aleph _{3,2}(x ,t) = \frac{{4{\hbar _2}^2}}{\varpi } - \frac{{12{\hbar _2}^2}}{\varpi }{\left( {\coth (\wp )} \right) ^2}. \end{aligned}$$

*Set 4* If ${k_0} = \frac{{1 + 2{\hbar _2}^2 - \frac{1}{{1 + 44{\hbar _2}^4}} - \frac{{2\mathrm{{i}}\sqrt{11} {\hbar _2}^2}}{{1 + 44{\hbar _2}^4}} - \frac{{44{\hbar _2}^4}}{{1 + 44{\hbar _2}^4}} - \frac{{88\mathrm{{i}}\sqrt{11} {\hbar _2}^6}}{{1 + 44{\hbar _2}^4}}}}{\varpi }$, ${k_1} = - \frac{{12\mathrm{{i}}{\hbar _2}^2}}{\varpi }$, ${k_2} = - \frac{{12{\hbar _2}^2}}{\varpi }$ and ${\hbar _1} = \frac{{{\hbar _2} + 2\mathrm{{i}}\sqrt{11} {\hbar _2}^3}}{{1 + 44{\hbar _2}^4}}$,then the periodic solution to Eq. (29) can then be derived as follows:

37 $$\begin{aligned} \begin{aligned} \aleph _{4,1}(x ,t) = \frac{{1 + 2{\hbar _2}^2 - \frac{1}{{1 + 44{\hbar _2}^4}} - \frac{{2\mathrm{{i}}\sqrt{11} {\hbar _2}^2}}{{1 + 44{\hbar _2}^4}} - \frac{{44{\hbar _2}^4}}{{1 + 44{\hbar _2}^4}} - \frac{{88\mathrm{{i}}\sqrt{11} {\hbar _2}^6}}{{1 + 44{\hbar _2}^4}}}}{\varpi } \\ - \frac{{12\mathrm{{i}}{\hbar _2}^2}}{\varpi }\tanh \left( \wp \right) - \frac{{12{\hbar _2}^2}}{\varpi }{\left( {\tanh (\wp )} \right) ^2}, \end{aligned} \end{aligned}$$

38 $$\begin{aligned} \begin{aligned} \aleph _{4,2}(x ,t) = \frac{{1 + 2{\hbar _2}^2 - \frac{1}{{1 + 44{\hbar _2}^4}} - \frac{{2\mathrm{{i}}\sqrt{11} {\hbar _2}^2}}{{1 + 44{\hbar _2}^4}} - \frac{{44{\hbar _2}^4}}{{1 + 44{\hbar _2}^4}} - \frac{{88\mathrm{{i}}\sqrt{11} {\hbar _2}^6}}{{1 + 44{\hbar _2}^4}}}}{\varpi } \\ - \frac{{12\mathrm{{i}}{\hbar _2}^2}}{\varpi }\coth \left( \wp \right) - \frac{{12{\hbar _2}^2}}{\varpi }{\left( {\coth (\wp )} \right) ^2}. \end{aligned} \end{aligned}$$

*Set 5* If ${k_0} = \frac{{ - {\hbar _1} + {\hbar _2} + 2{\hbar _1}{\hbar _2}^2}}{{{\hbar _1}\varpi }}$, ${k_1} = - \frac{{\sqrt{3} \sqrt{{\hbar _1}^2 - 2{\hbar _1}{\hbar _2} + {\hbar _2}^2 - 4{\hbar _1}^2{\hbar _2}^4} }}{{{\hbar _1}\varpi }}$ and

${k_2} = \frac{{{\hbar _1}^2 - 2{\hbar _1}{\hbar _2} + {\hbar _2}^2 - 4{\hbar _1}^2{\hbar _2}^4}}{{4{\hbar _1}^2\varpi {\hbar _2}^2}}$, then the following solution of Eq. (29) is obtained:

39 $$\begin{aligned} \begin{aligned} \aleph _{5,1}(x ,t) = \frac{{ - {\hbar _1} + {\hbar _2} + 2{\hbar _1}{\hbar _2}^2}}{{{\hbar _1}\varpi }} - \frac{{\sqrt{3} \sqrt{{\hbar _1}^2 - 2{\hbar _1}{\hbar _2} + {\hbar _2}^2 - 4{\hbar _1}^2{\hbar _2}^4} }}{{{\hbar _1}\varpi }}\tanh \left( \wp \right) \\ + \frac{{{\hbar _1}^2 - 2{\hbar _1}{\hbar _2} + {\hbar _2}^2 - 4{\hbar _1}^2{\hbar _2}^4}}{{4{\hbar _1}^2\varpi {\hbar _2}^2}}{\left( {\tanh (\wp )} \right) ^2}, \end{aligned} \end{aligned}$$

40 $$\begin{aligned} \begin{aligned} \aleph _{5,2}(x ,t) = \frac{{ - {\hbar _1} + {\hbar _2} + 2{\hbar _1}{\hbar _2}^2}}{{{\hbar _1}\varpi }} - \frac{{\sqrt{3} \sqrt{{\hbar _1}^2 - 2{\hbar _1}{\hbar _2} + {\hbar _2}^2 - 4{\hbar _1}^2{\hbar _2}^4} }}{{{\hbar _1}\varpi }}\coth \left( \wp \right) \\ + \frac{{{\hbar _1}^2 - 2{\hbar _1}{\hbar _2} + {\hbar _2}^2 - 4{\hbar _1}^2{\hbar _2}^4}}{{4{\hbar _1}^2\varpi {\hbar _2}^2}}{\left( {\coth (\wp )} \right) ^2}, \end{aligned} \end{aligned}$$

where $\hbar _{1}$, $\hbar _{2} \;and \;\varpi$ are the arbitrary parameters.

### Flowchart illustrating the two analytical methods

**Fig. 1:**
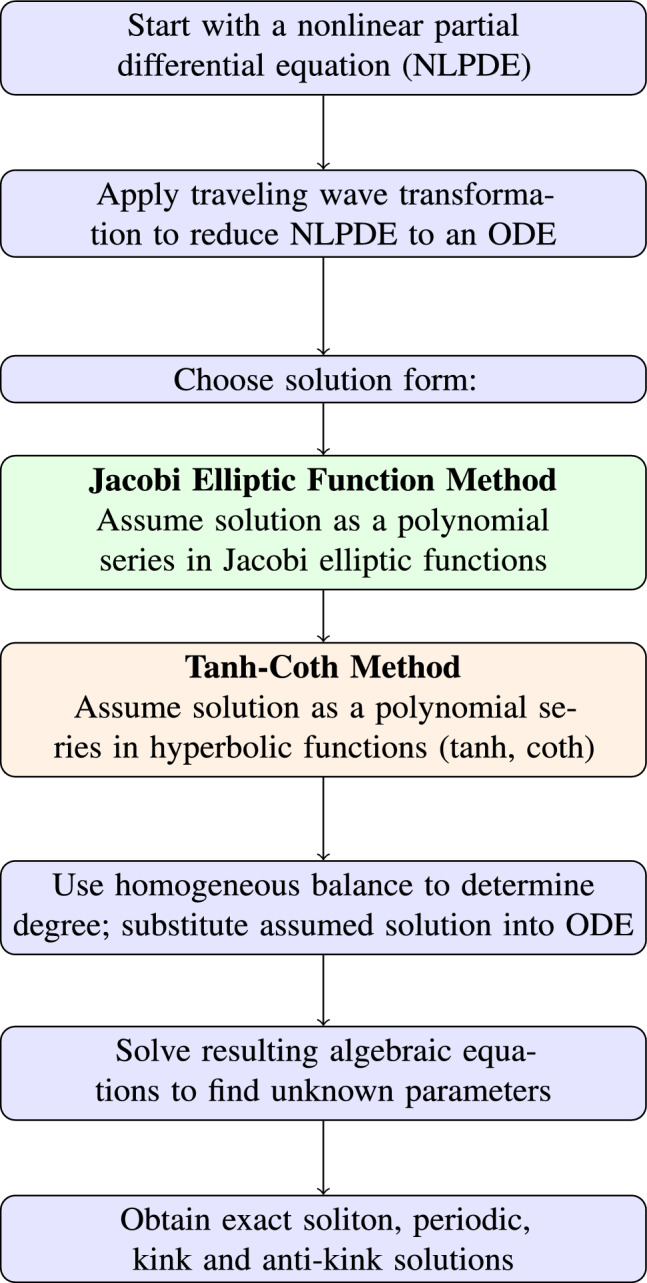
Flowchart summarizing the unified analytical procedure, highlighting both the Jacobi elliptic function method and the Tanh–Coth method.

To enhance the clarity of the solution process and help readers follow the methodological steps, Fig. 1 illustrates a combined flowchart of the two analytical methods used in this study: the Jacobi Elliptic Function approach and the Tanh–Coth method. This diagram systematically summarizes the main procedural steps involved in each technique, including the traveling wave transformation, the construction of solution ansätze, the balancing of nonlinear and derivative terms, and the derivation of exact soliton solutions. Presenting these steps visually not only improves the logical flow of the manuscript but also makes the solution strategies more accessible to readers, especially those new to analytical methods for fractional nonlinear partial differential equations.

## Results and discussion

**Fig. 2:**
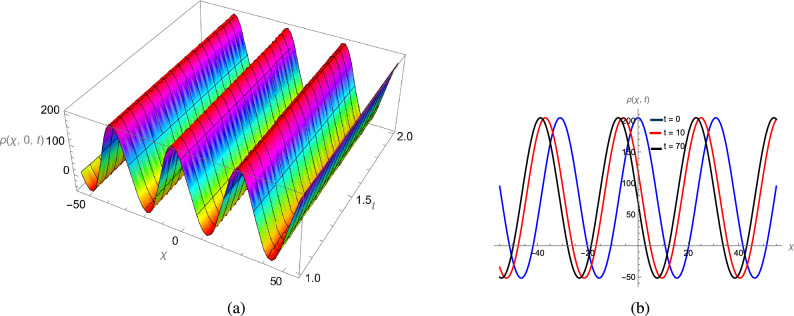
The TFJE Equation’s periodic pattern is associated with the solution ${\rho _{1,1}}(x,t)$ in Eq. (19) when $\varphi$ = 0.01, $\alpha$ = 1, $q = 0.7$, $\hbar _{1}$ = 0.09, $\hbar _{2}$ = − 10 and $\varpi$ = 1.5 are used.

**Fig. 3:**
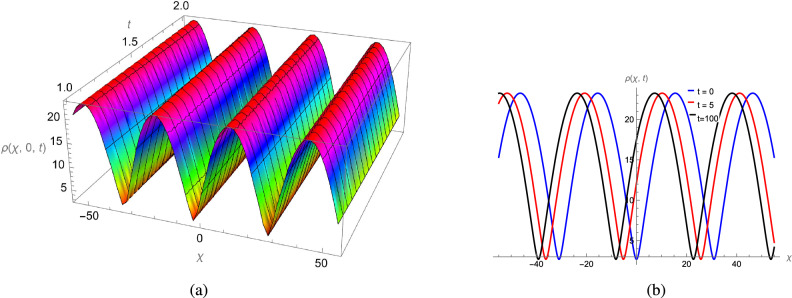
The TFJE Equation’s periodic pattern is associated with the solution ${\rho _{5,1}}(x,t)$ in Eq. (24) when $\varphi$ = 0.01, $\alpha$ = 1, $q = 0.8$, $\hbar _{1}$ = 0.09, $\hbar _{2}$ = − 10 and $\varpi$ = 1.5 are used.

**Fig. 4:**
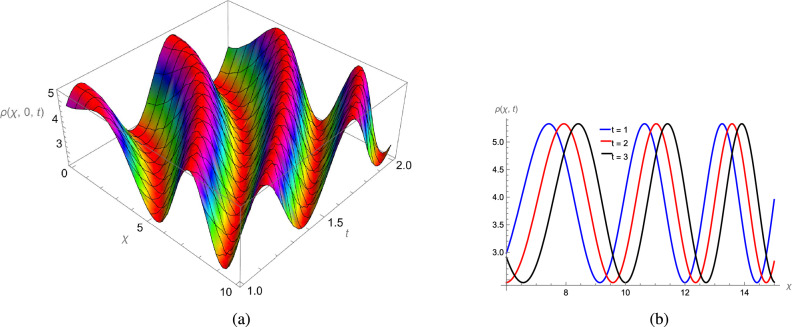
The TFJE Equation’s periodic profile for the solution ${\rho _{2,1}}(x,t)$ in Eq. (21) when $\varphi$ = 0.1, $\alpha$ = 1, $q = 0.1$, $\hbar _{1}$ = 0.09, $\hbar _{2}$ = 6 and $\varpi$ = 1.5 are used.

**Fig. 5:**
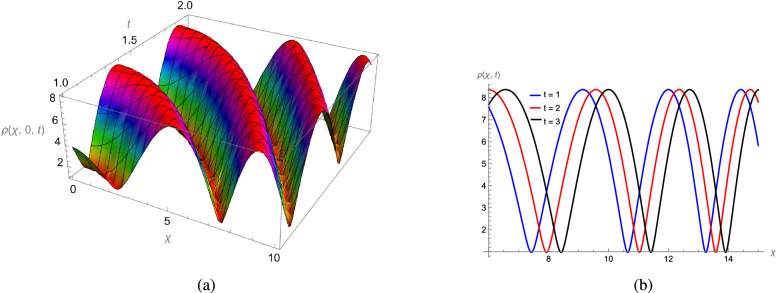
The TFJE Equation’s periodic profile for the solution ${\rho _{6,1}}(x,t)$ in Eq. (25) when $\varphi$ = 0.11; $\alpha$ = 1, $q = 0.1$, $\hbar _{1}$ = 0.09, $\hbar _{2}$ = 6 and $\varpi$ = 1.5 are used.

**Fig. 6:**
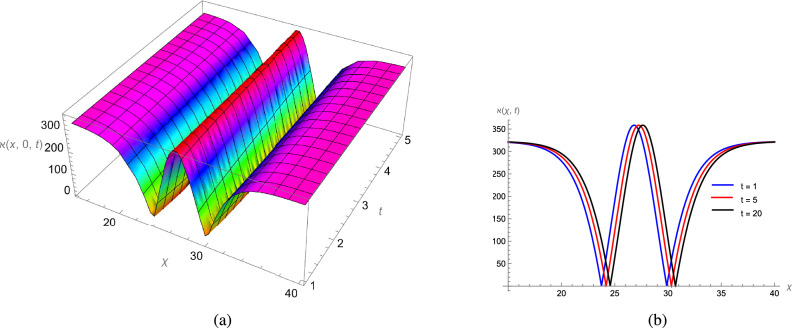
The W-shape profile of TFJE Equation for the solution ${\aleph _{1,1}}(x,t)$ in Eq. (31), for the values of $\hbar _{2}$ = 8, $\varphi$ = 0.01, $\alpha$ = 1, $\varpi$ = 1.5 and $\hbar _{1}$= 0.3.

**Fig. 7:**
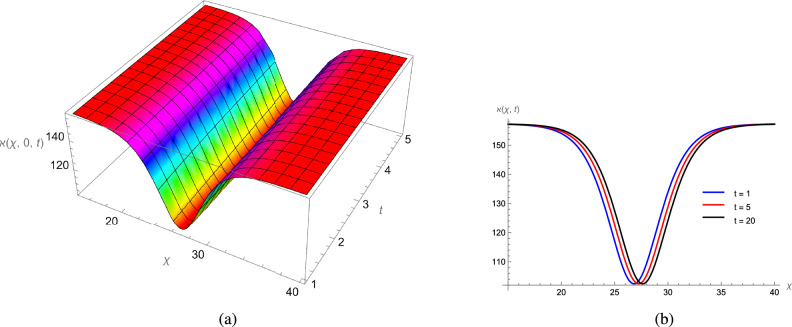
The anti bell shape profile of the TFJE Equation for the solution ${\aleph _{5,1}}(x,t)$ in Eq. (39) for the values of $\hbar _{2}$ = 8, $\varphi$ = 0.01, $\alpha$ = 1, $\varpi$ = 1.5 and $\hbar _{1}$ = 0.3.

**Fig. 8:**
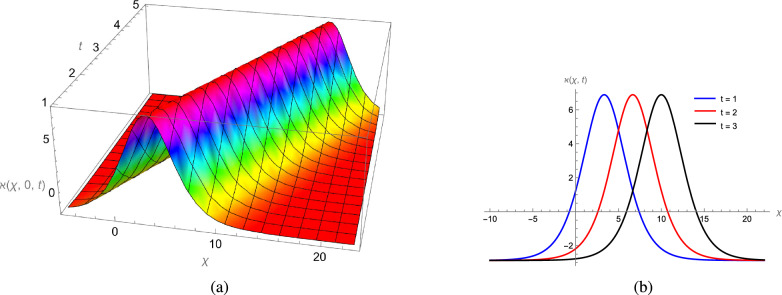
The bell shape profile of the TFJE Equation for the solution ${\aleph _{1,1}}(x,t)$ in Eq. (31) for the values of $\hbar _{2}$ = 1, $\varphi$ = 1, $\alpha$ = 1, $\varpi$ = 1.5 and $\hbar _{1}$ = 0.3.

**Fig. 9:**
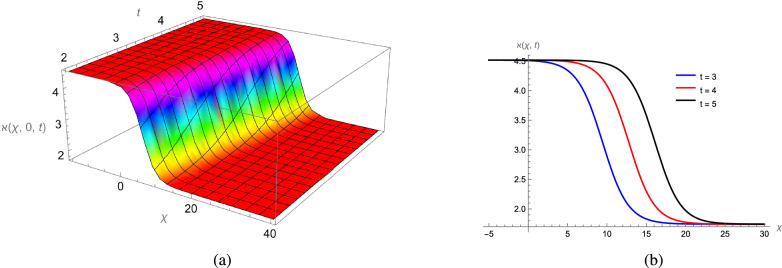
The smooth kink profile of the TFJE Equation for the solution ${\aleph _{5,1}}(x,t)$ in Eq. (39) for the values of $\hbar _{2}$ = 1, $\varphi$ = 1, $\alpha$ = 1, $\varpi$ = 1.5 and $\hbar _{1}$ = 0.3.

**Fig. 10:**
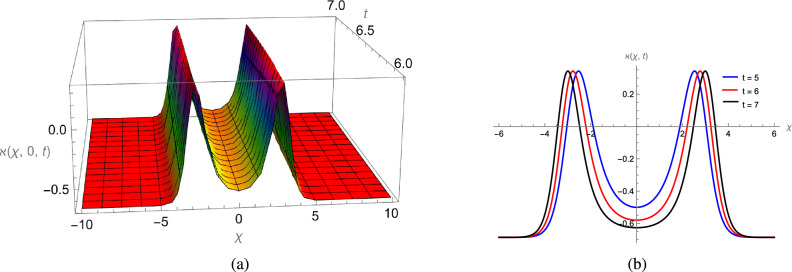
The Bell-shaped profile of the TFJE Equation for the solution ${\aleph _{3,1}}(x,t)$ in Eq. (35) for the values of $\hbar _{2}$ = 0.3,$\varphi$ = 1, $\alpha$ = 1 and $\varpi$ = 1.05.

**Fig. 11:**
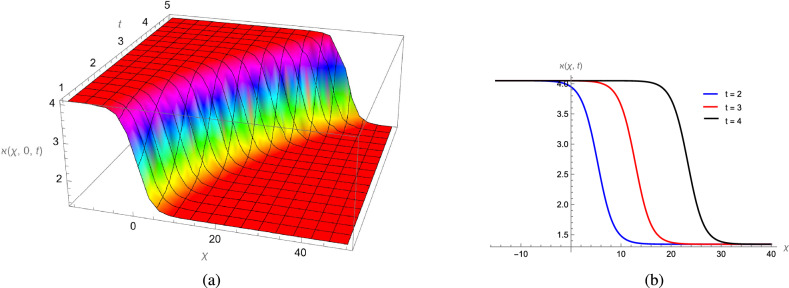
The kink profile of the TFJE Equation for the solution ${\aleph _{5,1}}(x,t)$ in Eq. (39) for the values of $\hbar _{2}$ = -0.9, $\varphi$ = 1, $\alpha$ = 1, $\varpi$ = 1.5 and $\hbar _{1}$ = 0.3.

In this study, we analytically solved the TFJE equation by applying Jumarie’s modified Riemann–Liouville derivative, which effectively captures the memory effects characteristic of fractional systems. Two analytical methods, the Jacobi Elliptic Function (JEF) method and the Tanh–Coth (TC) method, were employed to derive exact soliton solutions. These methods revealed diverse wave structures, including periodic solitons, bright solitons, W-shaped solitons, kink solitons, and dark solitons, each illustrating unique propagation behaviors influenced by fractional parameters. Figures 2, 3, 4 and 5 display periodic soliton solutions obtained via the JEF method. These solutions highlight the effect of parameters like the fractional orders $\varphi$ and $\alpha$, modulus *q*, and wave coefficients $\hbar _1$, $\hbar _2$, and $\varpi$ on wave shape and stability. For instance, increasing *q* from 0.7 to 0.8 (Fig. 3) produces solitons with shorter wavelength and higher oscillation frequency, while variations in $\varphi$ alter the smoothness and steepness of wave fronts. Figures 6, 7, 8, 9, 10 and 11 illustrate soliton types derived from the TC method. The W-shaped and dark solitons (Figs. 6, 7) show localized intensity dips, relevant in optical communication where they represent stable, non-dispersive data carriers. Bell-shaped and anti-bell-shaped solitons (Figs. 8, 10) reveal symmetric energy concentration, while kink solitons (Figs. 9, 11) show abrupt transitions, modeling wave fronts in nonlinear dispersive media. Overall, these results confirm the effectiveness of JEF and TC methods for constructing new families of exact solutions. The obtained figures emphasize how fractional parameters govern wave shape, width, and propagation dynamics, providing richer modeling of nonlinear wave phenomena compared to classical integer-order equations. This combination of analytical and graphical analysis offers deeper physical insight into the dynamics of the TFJE equation.

## Soliton solution’s novelty, advantage and limitations

The aim of this section is to contrast the solutions of the nonlinear TFJE equation obtained through the methods introduced in this paper with those from previous studies. In the work by Aylin Bayrak^62^, traveling wave solutions for the nonlinear TFJE problem were derived using the $(\frac{G^\prime }{G})$-expansion method with hyperbolic, trigonometric, and rational functions, applying the modified Riemann–Liouville fractional derivative. In contrast, our study identifies various soliton solutions—including periodic, kink, bell-shaped, anti-bell-shaped, and W-shaped solitons—using Jumarie’s modified Riemann–Liouville derivative in conjunction with JEF and TC methods. A comparison reveals that our results present novel solutions not covered in Bayrak’s publication, highlighting the distinctiveness and originality of our findings.

W-shaped solitons are localized wave patterns with two crests and a dip at the center, having a “W”-shaped profile. In practical applications, these solitons occur in different physical situations, mainly in nonlinear wave propagation. W-shaped solitons occur in nonlinear waveguides and optical fibers. They can model pulse propagation in systems with higher-order nonlinearity and dispersion, impacting signal stability in fiber optics. Periodic wave solitons are periodic solutions of nonlinear wave equations with repeated patterns in time or space. Periodic solitons are distinct from localized solitons (fading at infinity) in that periodic solitons oscillate repeatedly, creating wave-train-like patterns. A bell-shaped soliton is one form of a localized wave solution which travels and holds its form intact. While the periodic wave solitons repeat their cycles in both the spatial and time domains, solitons vanish at infinity without repeating their profiles. A kink soliton is a particular kind of soliton that is a topological wave. In contrast to bell-shaped solitons, which are localized pulses, kink solitons join two distinct stable states (asymptotic values) at $x\rightarrow \pm \infty$. These solitons play a critical role in domain wall models, phase transitions, and nonlinear wave propagation.

The nonlinear TFJE equation explains nonlinear wave propagation, especially for nonlinear dispersive media. Extending them to space-time fractional derivatives, the equations simulate physical systems with memory effects, anomalous diffusion, and nonlocal Space-time nonlinear TFJE equation simulate ultrashort optical pulses in nonlinear fiber optics. They enable the analysis of pulse broadening and self-phase modulation in the context of anomalous dispersion regimes. TFJE model ion-acoustic waves in plasma with nonlocal interaction. Applicable in the simulation of charged particle transport in magnetized plasmas, with memory effects playing a role on wave behavior. Space-time fractional models are useful for nonlinear transmission lines, representing the influence of fractional-order dispersion and damping. Applicable in telecommunication circuits where long-range interaction or memory effects take place. This equations provide a way of modeling nonlinear shallow water waves in fractional media. Applied in solitary wave and rogue wave prediction in oceanography. Fractional Joseph–Egri equations model the propagation of nerve impulses in biological systems with long-range memory effects. Applied in biomedical engineering to describe fractional diffusion in tissues. Space-time fractional models model the propagation of waves in cosmic plasmas as well as the dynamics of gravitational waves. Beneficial in the analysis of energy transport in astrophysical jets and plasma turbulence in space environment.

This study provides significant advantages by introducing novel soliton solutions for the time-fractional Joseph–Egri equation using the JEF and TC methods. The primary advantage is the ability to capture complex dynamics in fractional-order systems, which are difficult to model with traditional integer-order derivatives. However, a limitation of this approach lies in the potential complexity when extending these methods to higher-dimensional systems or more intricate nonlinear models. Despite this, the results offer a valuable framework for further exploration of fractional nonlinear partial differential equations in various applications.

## Conclusions

This study successfully applied the Jacobi Elliptic Function and Tanh–Coth methods to the time-fractional Joseph–Egri equation, deriving a broad spectrum of exact soliton solutions including periodic, bell-shaped, W-shaped, kink, and anti-bell-shaped waves. By leveraging Jumarie’s modified Riemann–Liouville derivative, the fractional dynamics of the equation were effectively captured, offering enhanced modeling of nonlinear wave propagation with memory effects. The graphical results emphasize the physical significance of the fractional order on soliton behavior. The originality of this work lies in expanding the known solution families for the fractional Joseph–Egri equation and demonstrating the robustness of these analytical methods for fractional nonlinear models. Future work may explore the application of these approaches to other fractional differential equations and real-world wave phenomena in physics and engineering. Additionally, it would be valuable to investigate the model using alternative definitions of fractional derivatives, such as the conformable fractional derivative, to further examine the influence of different fractional operators on soliton dynamics.

## Data Availability

Available from the corresponding author upon reasonable request
